# “Sarcopenia and risk of osteoporosis, falls and bone fractures in patients with chronic kidney disease: A systematic review”

**DOI:** 10.1371/journal.pone.0262572

**Published:** 2022-01-21

**Authors:** Anahita Rashid, Sabina Chaudhary Hauge, Charlotte Suetta, Ditte Hansen

**Affiliations:** 1 Department of Nephrology, Copenhagen University Hospital, Herlev and Gentofte, Denmark; 2 Geriatric Research Unit, Copenhagen University Hospital, Herlev and Gentofte, Denmark; 3 Geriatric Research Unit, Copenhagen University Hospital, Bispebjerg and Frederiksberg, Denmark; 4 CopenAge–Copenhagen Center for Clinical Age Research, University of Copenhagen, Denmark; 5 Department of Clinical Medicine, University of Copenhagen, Denmark; Medical College of Wisconsin, UNITED STATES

## Abstract

**Background:**

Chronic kidney disease [CKD] has been suggested to increase the risk of osteoporosis, sarcopenia, falls, and fractures. The aim of this systematic review was to explore the occurrence of osteoporosis, falls, and fractures in patients with sarcopenia and CKD, and to explore the possible association between sarcopenia and osteoporosis, falls, and fractures in patients with CKD.

**Methods:**

This systematic review was conducted according to the PRISMA guideline. The protocol was registered at PROSPERO. The systematic literature search was conducted in Pubmed [1966 to present] and EMBASE [1974 to present] on December 4, 2020. We searched for articles on CKD and sarcopenia, and then we selected them with outcomes such as osteoporosis, falls, and bone fractures. The risk of bias was assessed with the Newcastle-Ottawa Scale.

**Results:**

Five studies were eligible and included. No studies reported the occurrence of osteoporosis, falls, and bone fractures in patients with CKD and sarcopenia. Sarcopenia had a significant association with low bone mineral density [BMD] and osteoporosis in patients with CKD. The risk of bias assessed with the Newcastle-Ottawa Scale varied from 3–7 stars [median of 7]. Due to the included studies’ heterogeneity, a meta-analysis could not be conducted.

**Conclusion:**

The occurrence of osteoporosis, falls, and bone fractures in patients with sarcopenia and CKD could not be assessed from the included studies, but an association between sarcopenia and decreased BMD/osteoporosis in patients with CKD was found. The potential mechanistic link between sarcopenia and osteoporosis in CKD needs to be investigated in future studies.

## Introduction

Chronic kidney disease [CKD] is frequent, with a prevalence of around 13% globally [1]. Osteoporosis, falls, and fractures are common in CKD, and in end stage-kidney disease the risk of bone fracture is 2–3 times increased [2–4]. The precise mechanisms are not fully uncovered but the fragile bone is linked to changes in bone metabolism [5, 6]. There is also a disturbance in skeletal muscle mass in CKD due to increased protein catabolism and decreased anabolism [7], thereby potentially increasing the risk of sarcopenia in these patients. Sarcopenia was introduced in 1988 as an age-related decline in muscle mass [8]. It was recognized as a disease in 2016 with its ICD-10 code M62.84 [9]. The European Working Group on Sarcopenia [EWGSOP] [10] and other international groups [11, 12] have, over the years, developed numerous clinical definitions and cutoff values for sarcopenia, focusing on muscle mass, muscle strength, and muscle function. The prevalence of sarcopenia in the elderly population varies between 8–40%, which is most likely due to sarcopenia’s different diagnostic criterias [13–15]. Sarcopenia has been linked to poor outcomes such as osteoporosis [16], increased risk of falls [17], and fractures [18], which is suggested to be caused by a disturbed muscle-bone cross-talk [18]. The causes of Sarcopenia are multifactorial and sarcopenia is prevalent in patients with chronic diseases like CKD [19, 21], heart failure [20], rheumatoid arthritis [19], and malignancies [21]. The combination of CKD and sarcopenia, i.e. CKD-related sarcopenia, may develop more rapidly and typically occur earlier in adult life than age-related sarcopenia as a consequence of a negative energy-protein balance, insufficient diet and increased protein catabolism in many CKD patients [21].

Despite the negative health outcomes sarcopenia is not systematically investigated in CKD patients which is hampering effective treatments and the knowledge of CKD-related sarcopenia and its association with low bone quality and falls is limited. Fractures and falls lead to hospitalization, immobilization, and increased mortality in patients with CKD [16]. Hence it is of great interest to enhance our knowledge about the causes and prevalence of osteoporosis, falls and fractures in CKD-related sarcopenia. This systematic review aimed to investigate; 1 the occurrence of osteoporosis, falls, and bone fractures in patients with sarcopenia and CKD; 2 the possible association between sarcopenia and osteoporosis, falls or bone fractures in patients with CKD.

## Methods

This systematic review on CKD and the risk of osteoporosis, falls and bone fractures in patients with sarcopenia was conducted according to the PRISMA guideline [22]. The research questions were; “What is the occurrence of osteoporosis, falls, and bone fractures in patients with chronic kidney disease and sarcopenia?” and “Is sarcopenia associated with osteoporosis, falls, and bone fractures in patients with chronic kidney disease?” The protocol was registered at PROSPERO with registration number CRD42021242837.

### Literature search

The systematic literature search was conducted in Pubmed [1966 to present] and EMBASE [1974 to present]. The search strategy was discussed and developed in collaboration with a professional research librarian from the Copenhagen University Library. The search strategy was formed for PubMed and then adjusted to EMBASE and presented in the PROSPERO protocol [23]. In short, the search terms were related to sarcopenia, and CKD. The search was run in both databases on December 4, 2020. References were imported to [Mendeley Desktop 1.19.8] to remove duplicates, remaining duplicates were then manually removed, and the references were afterwards imported to Rayyan [Rayyan: Systematic Reviews version 2016] for screening.

### Study selection

According to our research questions, two reviewers screened the references separately based on titles and abstracts and later followed with the full-text screening in Rayyan. In case of conference abstracts, corresponding authors were contacted to inquire information about possible full-text versions. If we did not hear anything from the authors within fourteen days, we contacted them a second time. Finally, if they did not respond within seven days from the second contact date, we interpreted it as if only abstracts were available. A snowball search was also conducted by the two reviewers to find potentially relevant articles from the reference lists of the included studies. The inclusion criteria for relevant articles were studies with observational study design, which described patients ≥ 18 years, and included a population with CKD according to the definition by Kidney Disease: Improving Global Outcomes [24], and sarcopenia defined by decreased muscle mass and/or decreased muscle function or strength if investigated. Studies that reported the muscle mass from dual energy x-ray absorptiometry or bioimpedance analysis, muscle strength and quality in addition to functional testing or both in CKD patients were included. The studies also had to describe the occurrence of osteoporosis, falls, and/or bone fractures, or the association between sarcopenia and osteoporosis, falls and/or fractures in patients with CKD. Osteoporosis was defined according to the definition by World Health Organization [25] as reduced bone mineral density [BMD]. Studies describing falls or fractures either as prevalence or incidence could be included. Only articles written in English were included. Results only presented as abstracts were excluded. Conflicts concerning the inclusion of references were settled by consensus between the two reviewers.

### Data extraction

Data extracted from the included studies were entered into predefined tables. The data consisted of the journal, year, first author, title, country, study design, population, age, number of subjects, sarcopenia assessment method, the prevalence of sarcopenia, and data related to the occurrence of osteoporosis, falls or fractures, and the association between sarcopenia and either osteoporosis, falls or bone fractures in CKD. We contacted all five corresponding authors in addition to fifteen corresponding authors of relevant abstracts for additional data. None of the corresponding authors of the included studies responded to our inquiries and were contacted two times with 14 days apart. Nine corresponding authors of the relevant abstracts responded that no full text articles were available and could not present more data.

### Methodology quality assessment

The risk of bias was assessed with the Newcastle-Ottawa Scale [26], where the studies were graded with a score from zero to nine. A low number equaled a high risk of bias. A score of 5 or below was considered low quality, a score of 6 or 7 was considered moderate quality, and a score of 8 or 9 was considered high quality.

## Results

**Fig 1:**
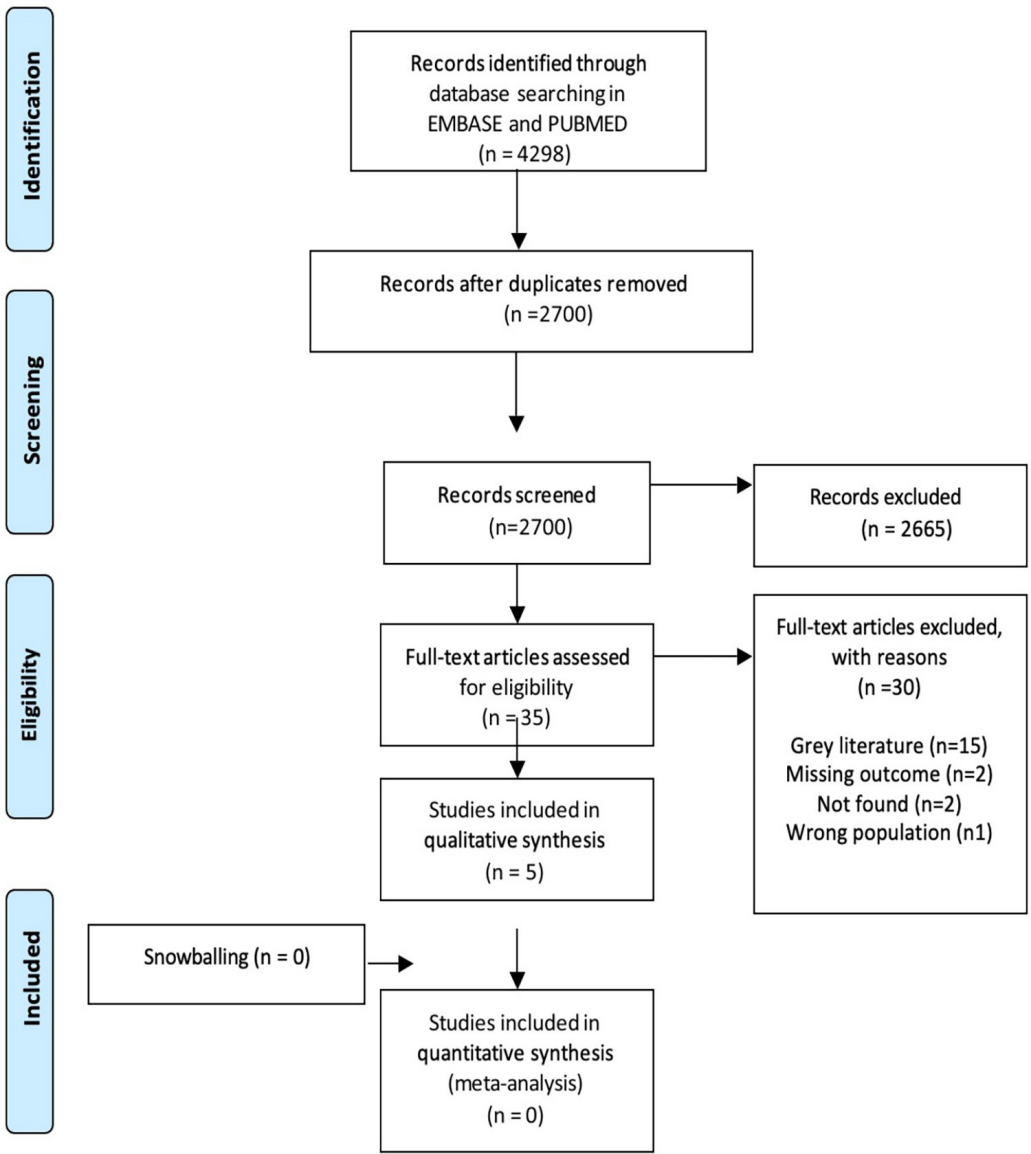
PRISMA Flow diagram. Depiction of the screening process.

The systematic search identified 2700 records after the removal of duplicates. After the screening process based on titles and abstracts; 2665 records were excluded. After assessing full-text articles, 6 studies were included in this review. The full selection process is depicted in Fig 1. This systematic review included 4 cross-sectional studies, and 1 retrospective observational study. The total number of subjects included was 763 participants.

Two studies reported results from patients on dialysis treatment [27, 28], two studies reported results from patients with CKD stage 3–4 [29, 30], and one study did not describe the applied definition of CKD [31]. Study characteristics of the included studies are presented in Table 1. We have displayed the nationality of the included studies in Table 1 rather than ethnicity since only one of the included studies [27] reported ethnicity with the majority of the population being black, except one white participant. All the studies were respectively conducted in South Korea, Japan or USA.

**Table 1:** Characteristics of included studies—osteoporosis, falls, and fracture.

Journal, Year, First author, Reference number	Country	Design	Population, Relevant subgroup	Age (years)	Subjects, Relevant subgroup, N	Sarcopenia assessment method	Prevalence of sarcopenia N, (%)	Osteoporosis	Falls	Bone Fracture	NOS score
Journal of Clinical Endocrinology and Metabolism, 2014, Yong-Ho Lee, [28]	South Korea	Cross-sectional study	≥ 50 years, CKD stage 3–4	25 (OH)D >20 ng/m L M: 70.4 ±7.7 F: 72.5 ± 7.1 25 (OH)D <20 ng/mL M: 71.1 ±7.8 F: 74.9 ± 7.4	6949, 571	Muscle Mass: ALM kg/weight	73 (13)	Sarcopenia has a significant association with osteoporosis and osteopenia	N/A	N/A	7
Osteoporosis International, 2014, J. E Kim, [29]	South Korea	Cross-sectional study	M and F>65 years, CKD stage 3	M: 73.5± 5.5 F: 74.2±6.3	2264, 80	Muscle Mass: ALM kg/height m^2^	8, (10)	The risk for sarcopenia was increased significant when CKD stage 3 and osteoporosis at the femur neck interacted.	N/A	N/A	7
J Bone Metabolism, 2018, Byung-Ho Yoon, [31]	South Korea	Retrospective study	F≥ 50 years with an osteoporotic fracture, CKD undefined	75.3±1.7	108, 9	Muscle Mass: ALM kg/height m^2^	6 (67)	The risk for sarcopenia was associated with osteoporotic fracture, but not statistically significant	N/A	N/A	3
Bone, 2019, Mary B. Leonard, [27]	USA	Cross-sectional study	Hemodialysis patients	48.4±10.3	29	Muscle Mass: ALM kg/height m^2^	N/A	ALM was associated with BMD in the total hip, femoral neck, ultradistal radius	N/A	N/A	7
Frontiers in Medicine, 2020, Kiyonori Ito, [28]	Japan	Cross-sectional study	Hemodialysis patients	69±10	50	Muscle mass: SMI Muscle strength: Handgrip strength	N/A	SMI was associated with the BMD of lumbar spine and femoral neck. Grip strength had a correlation with BMD, but it was not an independent factor.	N/A	N/A	7

CKD: Chronic kidney disease, M: Male, F: Female, SD: Standard deviation, N: number, kg: kilogram, N/A: not applicable, IQR: Interquartile range, 25(OH)D: 25-hydroxy-vitamin D, ALM: Appendicular Lean Mass, SMI: Skeletal Muscle Mass Index, BMD: Bone Mineral Density, NOS score: Newcastle-Ottawa Scale, BIA: Bioelectrical Impedance Analysis.

Generally, the studies differed due to heterogeneous designs and methodologies. The assessment methods for sarcopenia were diverse: 4 studies [27, 29–31] only used cutoff values for muscle mass to define sarcopenia, and 1 study [28] used both muscle mass and muscle strength to define sarcopenia. Three studies [29–31] reported the prevalence of sarcopenia to be between 10%-67%, with a median of 13%. Due to the included studies’ heterogeneity a meta-analysis could not be conducted. The heterogeneity was mainly due to differences in the study population, sarcopenia assessment methods, and data presentation related to outcomes.

### Occurrence of osteoporosis, falls and bone fractures

No eligible studies were found that investigated the prevalence or incidence of osteoporosis, falls, and bone fractures in patients with sarcopenia and CKD. Sarcopenia and association of osteoporosis, falls and bone fracture in patients with CKD.

### Sarcopenia and association of osteoporosis, falls and bone fracture in patients with CKD

In CKD stage 3–4 a significant association between osteoporosis and sarcopenia measured as appendicular skeletal mass was described in both of the two included studies [29, 30]. Two studies in patients on hemodialysis [27, 28] were included. They found an association between ALM and BMD [27], and an association between skeletal muscular index [SMI] measured by bioelectrical impedance analysis [BIA] and BMD [28]. No correlation between BMD and handgrip strength was found [28]. One study [31] did not describe the applied definition of CKD, but it investigated the association between CKD, and sarcopenia in patients with osteoporotic fractures and found no significant association. None of the studies described the association between sarcopenia and falls or bone fractures in patients with CKD.

### Quality assessment of the individual studies

The risk of bias assessed with the Newcastle-Ottawa Scale varied from 3–7 stars [median of 7 stars] across the included studies depicted in Table 1. Except for one study [31], the included studies were considered moderate to high quality.

## Discussion

This systematic review aimed to explore the occurrence of osteoporosis, falls, and/or bone fractures in patients with sarcopenia and CKD, and it also aimed to explore the association between sarcopenia and osteoporosis, falls, and/or fractures in patients with CKD. Five studies were included with a total of 739 participants. Whereas the association between sarcopenia and decreased BMD/osteoporosis was described, the occurrence of osteoporosis, falls, and bone fractures in patients with sarcopenia and CKD could not be determined from the included studies, nor were the association between sarcopenia and falls or fractures reported. Only one study [28] used both muscle mass and muscle strength to define sarcopenia, this is in contrast to the EWGSOP recommendations [10] which also, in addition to muscle mass and muscle strength, includes parameters for muscle function to define sarcopenia.

Overall, an association between sarcopenia measured as either ALM by dual energy x-ray absorptiometry [DXA] [27, 29, 30] or as skeletal muscle mass index [SMI] by BIA [28] and osteoporosis determined by BMD was found. Two of the included studies found an increased risk of sarcopenia in CKD patients with osteoporosis compared to patient without osteoporosis [29, 30]. The association between sarcopenia and decreased BMD or osteoporosis was seen at all stages of CKD. This supports the hypothesis that CKD-related sarcopenia begins in the early course of kidney disease [21]. It must be considered that one of the included studies did not support the association between sarcopenia and osteoporosis in patients with CKD [31]. The retrospective study did not find any association between CKD and ALM. However, the subpopulation of CKD was very small, which may be too small to detect any impact of CKD [31] Furthermore, the methodology of the study was sparsely described and it was graded as of only low quality. HR-pQCT is a new diagnostic tool to evaluate bone microarchitecture and it is able to differentiate between cortical and trabecular bone. Cortical porosity assessed by HR-pQCT associates with increased prevalence of fractures in hemodialysis patients [32], but the prognostic value on fracture risk in CKD is still to be determined. In peritoneal dialysis ALM has been found positively associated with trabecular number [33]. If the reduced BMD in the patients with CKD-related sarcopenia is due to reduced trabecular bone remains to be further explored.

Muscle mass measured as both ALM by DXA and SMI by BIA showed an association with decreased BMD and osteoporosis. Only one study measured the muscle strength in terms of hand grip strength as an indicator of sarcopenia. Hand grip strength was not found to have an independent influence on BMD [28]. It may be speculated that the muscle strength in the lumbar spine or femur affects the regional BMD at the femur neck and/or lumbar spine, and thereby is of greater importance for the regional BMD than the hand grip strength. Therefore, a potential association could be hard to find in this specific design. Nevertheless, in the revised sarcopenia consensus on sarcopenia from the EWGSOP [10], handgrip strength is presented to correlate moderately with strength in other body compartments. It is seen as a reliable surrogate for more complicated measures of arm and leg strength. Hence an association between handgrip strength and BMD was expected. No studies were found that used muscle strength in addition to functional testing. Hopefully this tendency will change and more studies will include these aspects in the definition of sarcopenia.

Osteoporosis is common in patients with sarcopenia. In a systematic review and meta-analysis [34] the prevalence of osteoporosis was 5–37% in populations with sarcopenia, depending on the characteristics of the studied population and the used definition of sarcopenia. Osteoporosis is also frequently found in patients with CKD [35, 36], and an increased prevalence of osteoporosis in patients with CKD and sarcopenia was expected. Unfortunately, the included studies did not report the frequency of osteoporosis in patients with sarcopenia and CKD, and further studies should be performed to address the size of this important clinical problem.

CKD is well-known to increase the risk for accidental low-energy falls and is associated with morbidity and mortality [37]. In a systematic review, the risk of falls in CKD patients ranged between 1.18 and 1.60 falls/patient year. The falls were frequent in the older patients on hemodialysis, and recurring falls caused serious consequences such as death, limited mobility, hospitalizations, and admissions to nursing homes [38]. In a systematic review and meta-analysis, patients with sarcopenia also had a higher risk of accidental falls and fractures [39]. As such, patients with CKD-related sarcopenia may be at high risk of falls. Unfortunately, no eligible studies were found in the systematic search which could address this question. The fracture risk was not reported in the included studies. The fracture risk is known to be higher in patients with CKD and in patients with sarcopenia. In Hansen et al. [3], the fracture risk was significantly higher in a CKD-population on kidney replacement therapy. Specifically, the hazard ratio for any fracture was 3.14 [95% CI 2.97–3.31] in the dialysis subpopulation. In a systematic review and meta-analysis [34], the risk of fracture was 1.37 [95% Cl 1.18–1.59] in a sarcopenic population. A cross-sectional study [40], published after our literature search, explored the prevalence of history of bone fracture and sarcopenia defined as SMI measured by BIA in predialysis CKD patients. An increased history of bone fracture was found in patients with low estimated glomerular filtration rate and low skeletal muscle mass. The odds ratio was 1.15 [95% CI 1.03–1.29] for a history of bone fracture for every 1 kilogram decrease in skeletal muscle mass. Based on these findings, the fracture risk is expected to be high in patients with CKD and sarcopenia. This is supported by the findings in this review of a significant relationship between low BMD and sarcopenia in CKD patients. Osteoporosis and sarcopenia may interact through different pathways in patients with CKD. Disturbances in skeletal muscle mass in CKD may be due to increased protein catabolism and decreased anabolism [7], thereby potentially increasing the risk of sarcopenia in these patients. The disturbances in skeletal muscle may be problematic because sarcopenia may contribute to the development of osteoporosis, potentially by an inhibition of muscle contraction causing inhibition of osteogenesis, hypothesized in the mechanostat hypothesis [41]. Cross-talk in the ’bone-muscle’ unit is also thought to be important as a site where the muscle and bone tissue communicate via paracrine and endocrine pathways to regulate their development [18]. Classification of these pathways that affect both bone and muscle may facilitate the development of new treatments for both osteoporosis and sarcopenia in CKD patients. Vitamin D seems to be associated with BMD in patients with sarcopenia and CKD, as CKD patients with vitamin D deficiency have an increased risk of osteoporosis or osteopenia that is also associated with sarcopenia [29]. Vitamin D plays a vital role in bone and mineral metabolism in CKD and has been significantly associated with sarcopenia [10]. The influence of vitamin D on CKD-related sarcopenia should be further studied.

Renal osteodystrophy encompasses a spectrum of various changes in bone pathology including low and high bone turnover and osteomalacia [6]. All of these pathologies may lead to reduced BMD and increased risk of fracture. The relation between specific types of renal osteodystrophy and presence of sarcopenia is unknown. However, in a recent study of Australian dialysis patients [42] no association between sarcopenia and parathyroid hormone, one of the best non-invasive markers of bone turnover, was found.

Obesity may increase BMD and the coexistence of sarcopenia and obesity could influence on the risk of osteoporosis in CKD [21]. None of the studies examined this association and this should be further explored in future studies. BMI were adjusted for in the analysis in two of the studies [29, 30].

Currently, no pharmaceutical treatment of sarcopenia exists, but potential therapies such as myostatin antibodies are being investigated in elderly [43]. Exercise interventions have already shown to have a positive effect on the musculoskeletal system in elderly and CKD [44, 45]. This systematic review has several strengths. The search strategy was developed with a research librarian, and the search was performed in two databases, Pubmed and EMBASE, on the same date. A supplemental snowball-search was also conducted to identify possible relevant studies. According to the PRISMA guideline [22], a PROSPERO was registered [23]. The bias in the studies was assessed using the recommend Newcastle-Ottawa Scale for observational studies [26]. Nevertheless, this review was also limited due to the small number of eligible studies and to the heterogeneity in the included studies; therefore, a meta-analysis was not possible. Despite the heterogeneity in the studies the data was presented systematically to present the studies and the findings that are currently available and the overall tendencies and to inform about this gap in the literature. Many potential eligible studies were never published as full-text versions and were only available as conference abstracts, and they were therefore excluded in this review. The operational definition of sarcopenia and the reporting of outcomes differed considerably across the studies. The lack of a universal definition to determine sarcopenia makes it challenging to evaluate and compare data. We would also have included studies that used muscle strength and quality in addition to function testing, but these studies were not found in our search in CKD-populations. It seems that the focus in sarcopenia studies in CKD-studies still lies on muscle mass despite the EWGSOP criteria of sarcopenia. Hopefully this tendency will change and more studies will use the EWGSOP criteria.

Unfortunately, the included studies did not report the occurrence of osteoporosis, falls, and bone fractures in patients with sarcopenia and CKD, but tended to investigate the associations between osteoporosis and sarcopenia in various correlation analyses. Therefore, there is a need for a more detailed description of the occurrence of osteoporosis, falls, and fractures in patients with CKD and sarcopenia. In addition to studies that investigate pathophysiological links between bone and muscle of sarcopenia in CKD, there is also a need to investigate preventive and therapeutic interventions for these patients.

## Conclusion

This systematic review aimed to investigate the occurrence of osteoporosis, falls, and bone fractures in patients with sarcopenia and CKD, and the association between sarcopenia and osteoporosis, falls, and fractures in patients with CKD. Six studies were included with a total of 763 participants. The prevalence of osteoporosis, fall, and bone fractures could not be described from the included studies, but an association between sarcopenia and osteoporosis/a decreased BMD in patients with CKD 3-5D was found.

## Supporting information

S1 Checklist Prisma flowchart. (DOCX)

S1 Table Cutoff-off points used for sarcopenia assessment and detailed quality assessment. (DOCX)

S1 File Search string PUBMED. (DOCX)
