# Supplementary material for: “Sarcopenia and risk of osteoporosis, falls and bone fractures in patients with chronic kidney disease: A systematic review”
Source: PLoS One. 2022 Jan 21;17(1):e0262572. doi: 10.1371/journal.pone.0262572 (PMC8782402; doi:10.1371/journal.pone.0262572)
Supplement: S1 Table — (DOCX) [file pone.0262572.s002.docx]

| **Journal,**  **Year,**  **First author** | **Sarcopenia assesment method** | **Newcastle Ottawa scale – detailed scores** |
| --- | --- | --- |
| Journal of Clinical Endocrinology and Metabolism, 2014,  Yong-ho Lee | DXA Appendicular skeletal muscle mass, (kg/weight), less than 2 SD below the sex-specific mean for a young reference group, modified from Janssen et al.  Cutoff value for sarcopenia was 29.0% for males and 22.9% for females. | Selection: 4  Comparability: 2  Outcome: 1  Total score 7 |
| Osteoporosis International,  2014,  J. E. Kim | Sarcopenia was defined as an appendicular skeletal muscle mass divided by height^2^ of less than 1 standard deviation below the sex-specific mean for a younger reference group aged 20–39 years. The cutoff value for sarcopenia was 7.04 kg/m^2^ for men and 5.04 kg/m^2^ for women. | Selection: 4  Comparability: 2  Outcome: 1  Total score 7 |
| J Bone Metabolism,  2018, Byung-Ho Yoon | DXA Appendicular skeletal mass, kg/m^2,^ cutt off values from the Asian Working Group for Sarcopenia (7.00 kg/m^2^ for men and 5.40 kg/m^2^ for women). | Selection: 1  Comparability: 1  Outcome: 1  Total score: 3 |
| Bone,  2019,  Mary B. Leonard | DXA appendicular lean mass index (ALMI kg/m^2^) Z-scores were significantly lower in the hemodialysis patients than the healthy DXA controls. | Selection: 4  Comparability: 2  Outcome: 1  Total score 7 |
| Frontiers in Medicine,  2020, Kiyonori Ito | BIA Skeletal muscle mass index (kg/m^2^) and grip strength (kg)  The SMI in all patients was 6.10 ± 1.20 kg/m2, and the grip strength was 23 ± 8 kg. | Selection: 4  Comparability: 2  Outcome: 1  Total score 7 |
