# Supplementary material for: “Sarcopenia and risk of osteoporosis, falls and bone fractures in patients with chronic kidney disease: A systematic review”
Source: PLoS One. 2022 Jan 21;17(1):e0262572. doi: 10.1371/journal.pone.0262572 (PMC8782402; doi:10.1371/journal.pone.0262572)
Supplement: S1 File — (DOCX) [file pone.0262572.s003.docx]

**BLOCK1 Keywords**

- **"muscular atrophy"[Mesh])**
- **"muscle loss*”**
- **"skeletal muscle mass*"**
- **"diminished strength*"**
- **"muscularit*"**
- **"muscle protein degradation*"**
- **“muscle protein breakdown*”**
- **"lean body mass*"**
- **"lean soft tissue*"**
- **"muscle atroph*”**
- **"sarcopen*”**
- **"low muscle quantit*"**
- **"muscle wasting*"**
- **"low muscle qualit*"**
- **"poor physical performance*"**
- **"mobility disorder*"**
- **"low muscle strength*"**
- **"low physical performance*"**
- **"appendicular lean mass*"**

**BLOCK2 Keywords**

- **"renal insufficiency, chronic"[Mesh]**
- **"end-stage kidney*"**
- **"ESRF”**
- **"ESKF"**
- **"ESRD”**
- **"ESKD"**
- **"end-stage renal*"**
- **"CKF"**
- **"CKD"**
- **"chronic kidney*"**
- **"chronic renal*"**
- **"irreversible kidney*"**
- **"irreversible renal*"**
- **“nephropath*”**
- **"renal replacement therap*"**
- **“kidney replacement therap*”**
- **"KFRT"**
- **“KRT”**
- **“RRT”**
- **"dialysis"**
- **"hemodialysis"**
- **"haemodialysis"**
- **"hemofiltrat*"**
- **"haemofiltrat*"**
- **"hemodiafiltrat*"**
- **"haemodiafiltrat*"**
- **"HF dialysis"**
- **"HDF dialysis"**
- **"peritoneal dialysis"**
- **"CAPD"**
- **"CCPD"**
- **"APD"**

# Search string EMBASE

**BLOCK1 Keywords**

**●exp muscle atrophy/ or exp sarcopenia/**

**●"muscle loss*”**

**●"skeletal muscle mass*"**

**●"diminished strength*"**

**●"muscularit*"**

**●"muscle protein degradation*"**

**● “muscle protein breakdown*”**

**●"lean body mass*"**

**●"lean soft tissue*"**

**●"muscle atroph*”**

**●"sarcopen*”**

**●"low muscle quantit*"**

**●"muscle wasting*"**

**●"low muscle qualit*"**

**●"poor physical performance*"**

**●"mobility disorder*"**

**●"low muscle strength*"**

**●"low physical performance*"**

**●"appendicular lean mass*"**

**BLOCK2 Keywords**

**●exp chronic kidney failure/ or exp kidney failure/ or exp "chronic kidney disease-mineral and bone disorder"**

**●"end-stage kidney*"**

**●"ESRF”**

**●"ESKF"**

**●"ESRD”**

**●"ESKD"**

**●"end-stage renal*"**

**●"CKF"**

**●"CKD"**

**●"chronic kidney*"**

**●"chronic renal*"**

**●"irreversible kidney*"**

**●"irreversible renal*"**

**● “nephropath*”**

**●"renal replacement therap*"**

**● “kidney replacement therap*”**

**●"KFRT"**

**● “KRT”**

**● “RRT”**

**●"dialysis"**

**●"hemodialysis"**

**●"haemodialysis"**

**●"hemofiltrat*"**

**●"haemofiltrat*"**

**●"hemodiafiltrat*"**

**●"haemodiafiltrat*"**

**●"HF dialysis"**

**●"HDF dialysis"**

**●"peritoneal dialysis"**

**●"CAPD"**

**●"CCPD”**
